# Advances and Challenges in Electrolyte Development for Magnesium–Sulfur Batteries: A Comprehensive Review

**DOI:** 10.3390/molecules29061234

**Published:** 2024-03-11

**Authors:** Lin Sheng, Junrun Feng, Manxi Gong, Lun Zhang, Jonathan Harding, Zhangxiang Hao, Feng Ryan Wang

**Affiliations:** 1 School of Mechanical and Electronic Engineering, Suzhou University, Suzhou 234000, China; shenglin@ahszu.edu.cn; 2 School of Science, School of Chip Industry, Hubei University of Technology, Wuhan 430068, China; 3 Materials and Catalysis Laboratory, Department of Chemical Engineering, University College London, London WC1E 7JE, UK; m.gong@ucl.ac.uk (M.G.); lun.zhang.19@ucl.ac.uk (L.Z.); 4 Department of Electrical Engineering and Electronics, University of Liverpool, Liverpool L69 3GJ, UK; jonathan.harding2@liverpool.ac.uk

**Keywords:** magnesium–sulfur, battery, electrolyte, non-nucleophilic, nucleophilic

## Abstract

Magnesium–sulfur batteries are an emerging technology. With their elevated theoretical energy density, enhanced safety, and cost-efficiency, they have the ability to transform the energy storage market. This review investigates the obstacles and progress made in the field of electrolytes which are especially designed for magnesium–sulfur batteries. The primary focus of the review lies in identifying electrolytes that can facilitate the reversible electroplating and stripping of Mg^2+^ ions whilst maintaining compatibility with sulfur cathodes and other battery components. The review also addresses the critical issue of managing the shuttle effect on soluble magnesium polysulfide by looking at the innovative engineering methods used at the sulfur cathode’s interface and in the microstructure design, both of which can enhance the reaction kinetics and overall battery efficiency. This review emphasizes the significance of reaction mechanism analysis from the recent studies on magnesium–sulfur batteries. Through analysis of the insights proposed in the latest literature, this review identifies the gaps in the current research and suggests future directions which can enhance the electrochemical performance of Mg-S batteries. Our analysis highlights the importance of innovative electrolyte solutions and provides a deeper understanding of the reaction mechanisms in order to overcome the existing barriers and pave the way for the practical application of Mg-S battery technology.

## 1. Introduction

The successful application of rechargeable Li-ion batteries (LIBs) has lead to the dramatic development of portable electronic devices, electric vehicles, and large-scale energy storage systems in recent decades as the world attempts to tackle increasingly intensive climate and environmental problems [1,2,3,4]. However, LIBs are failing to catch up with the ever-growing safety and energy density demands of emerging applications, such as electric vehicles with long endurances and unmanned aerial vehicles [3,5,6]. The high price of the corresponding materials for LIBs also introduces concerns with the requirement for new rechargeable battery systems to have an economical cost alongside their high-energy density to achieve a sustainable future [7,8,9,10]. In recent decades, magnesium batteries have attracted growing interest as a promising candidate for post-lithium-ion battery systems [11,12,13,14,15]. Divalent Mg^2+^ enables two electron transfers per Mg atom, resulting in a high theoretical specific capacity of 2205 mAh g^−1^ [16,17,18]. Considering the density of magnesium, the Mg anode enables a high volumetric capacity of 3833 mAh mL^−1^, which is almost double that of lithium (2062 mAh mL^−1^) [17,19,20,21,22]. Moreover, a Mg anode displays advantages such as low cost, high Earth abundance, and easy operation under air [23,24,25].

In spite of these advantages, the use of Mg as an anode in rechargeable batteries is still hampered by the limited choice of electrolytes and cathode materials in view of facile passivation of the Mg’s surface and the sluggish solid-state diffusion of the highly polar divalent cations in the lattices [26,27,28,29,30,31]. Up until now, pure Mg-ion systems based on the intercalation mechanism have had a limited energy density, usually less than 300 Wh kg^−1^ without counting the weight of the solvent [32,33,34]. Therefore, it is highly desirable to develop novel and safe systems and materials based on Mg^2+^/Mg metal conversion chemistry with a higher energy density, where anode dendrite growth can be effectively suppressed [35,36,37]. Sulfur is one of the most promising candidates for conversion cathodes because of its high theoretical capacity (1672 mAh g^−1^) and volumetric capacity (3459 mAh mL^−1^), as well as its reserve abundance [38,39,40,41]. Sulfur cathodes have been well developed in Li-S battery systems. The cell exhibits a high theoretical volumetric energy density of 2856 Wh kg^−1^, which is over four times higher than that of LIBs [42,43]. However, Li-S batteries suffer from poor long-term stability due to the formation of unwanted solid electrolyte interphases (SEIs), shuttle effects, and the growth of lithium dendrites during their operation [44,45,46]. Compared to the intensive research process for Li-S batteries, Mg-S batteries show a higher volumetric capacity of 3221 mAh mL^−1^ but they are still in their nascent stage [19,47]. Development has been impeded due to the numerous issues present in the system. Firstly, suitable electrolytes are required. Commonly used Mg-ion battery electrolytes such as magnesium perchlorate acetonitrile are nucleophilic in nature and therefore cannot support the reversible Mg-ion redox reaction in a Mg-S battery [23]. Numerous efforts have been made to find compatible electrolytes for the system. For example, Muldoon et al. reported on a Hauser base electrolyte that is produced according to the reaction of AlCl_3_ with hexamethyldisilazide magnesium chloride (HMDSMgCl) in a tetrahydrofuran (THF) solvent to form a non-nucleophilic [Mg_2_(μ-Cl)_3_6THF][HMDSAlCl_3_] complex, which showed a high voltage stability of up to 3.2 V [48]. Furthermore, electrolyte additives such as salts, ethereal solvents, and ionic liquid additives have been used to improve the performance of the electrolyte used by Muldoon et al. above [49,50,51]. Non-nucleophilic electrolytes are pivotal in augmenting the stability and efficiency of Mg-S batteries. Researchers have developed both chloride-inclusive and chloride-exempt non-nucleophilic electrolyte systems. For example, chloride-inclusive hexamethyldisilazane magnesium chloride (HMDSMgCl)-based non-nucleophilic electrolytes have exhibited favorable compatibility with sulfur cathodes [52]. Moreover, notwithstanding the typical incompatibility of nucleophilic organic magnesium compounds with sulfur-based conversion cathodes, scholars have effectively modified nucleophilic (PhMgCl)_2_-AlCl_3_/THF electrolytes to conform to the needs of Mg-S batteries [53,54]. These systems benefit from their similarity to the sulfur reduction mechanism in Li-S batteries, which means it is possible to try to use the same base cathode materials [44,55,56,57], electrolytes [58,59,60], separators [61,62,63], and methodologies [64,65] for the development of Mg-S batteries.

The purpose of this review is to summarize the most up-to-date understanding in the field of electrolytes for Mg-S batteries, highlighting the typical methods and examples that have contributed to the development of these electrolytes. Furthermore, since the research into Mg-S batteries is still in its early stages, the principle of Mg-S batteries will also be discussed, as it has not yet been analyzed in the results of a definitive study. Finally, the remaining challenges and future perspectives will be given in an attempt to inspire researchers in this area.

## 2. Principle of Mg-S Batteries

**Figure 1:**
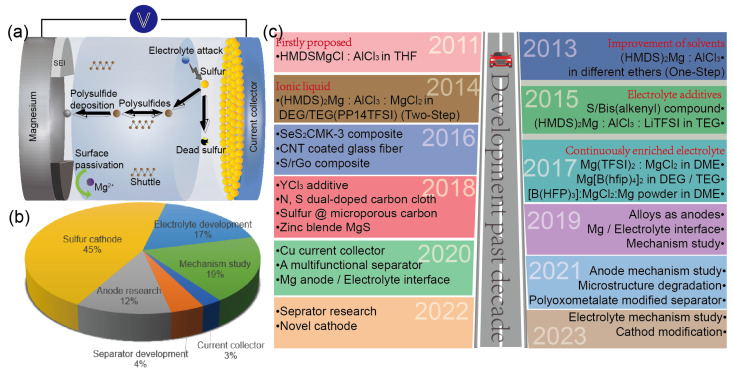
(**a**) Structure and working principles of Mg-S battery; (**b**) proportional representation of research directions, including electrolytes, sulfur-based cathodes, magnesium anodes, separators, current collectors, and mechanistic studies; (**c**) significant advancements in the Mg-S battery field since 2011.

The construction of a Mg-S battery (Figure 1a) comprises a magnesium anode, a sulfur cathode, and electrolytes, which together illustrate the structure and working principle of the battery. The magnesium atom loses two electrons during discharge, becomes Mg^2+^ at the anode, and dissolves in the electrolyte. The Mg^2+^ migrates through the cell separator and reaches the sulfur on the electrolyte’s cathode side surface [66]. The electrons then proceed to the sulfur cathode through an external connection, where elemental sulfur generates electrons. In the sulfur cathode’s continuous reduction process, elemental sulfur is transformed into long-chain magnesium polysulfide (MgS_x_, 4 ≤ x ≤ 8), which dissociates into short-chain magnesium polysulfide (MgS_x_, 2 ≤ x < 4). This short-chain magnesium polysulfide is then transformed into magnesium sulfide (MgS) [55,67,68,69]. The external circuit applies a current or voltage to the Mg-S battery during the charging process. In the MgS, S^2−^ loses electrons and transforms into elemental sulfur, followed by the dissolution of Mg^2+^ in the electrolyte. The magnesium ions in the electrolyte migrate from the cathode to the magnesium anode, driven by the current, which completes the Mg-S battery’s discharge/charge cycle. In the anode of magnesium, Mg is oxidized during the discharging process. The cathode is restored during the charging process to complete the battery’s internal migration and forms a complete battery path [26,56,70]. The main equations for the reactions are as follows:

Magnesium anode: Mg − 2e^−^ = Mg^2+^,

Sulfur cathode: S + 2e^−^ + Mg^2+^ = MgS,

Total response: Mg + S = MgS.

So far, the research on Mg-S batteries has focused on the cathode, anode, and electrolyte (Figure 1b). The materials of the anode and cathode in Mg-S batteries include the following types:

Anode: Mg metal is the most commonly used anode material for Mg-S batteries; depending on the type of battery, its form may vary between foils [17,67,71,72], plates [51,73], and discs [74,75,76,77]. In addition to Mg metal, Mg–carbon composite materials are also used as anode modifiers [55,78,79]. The carbon provides the anode with good electronic conductivity and a high surface area, meaning a higher capacity and current rate capacity [80].

Cathode: The material of the cathode is a primary research priority for Mg-S battery performance enhancement [81]. The composition of the cathode materials mainly includes S/carbon and S/MOF. The role of the carbon [17,52,82,83,84] and Metal–Organic Frameworks (s) [72] in the cathode is mainly that of a conductive agent, due to the low electrical conductivity of sulfur. Since 2014, when Zhao-Karger et al. used CMK-3 [55], a mesoporous carbon material, as the conductive agent and as a container to fix the sulfur and mitigate the shuttle effect [85], increasing numbers of researchers are now implementing mesoporous-structure conductive agents in the cathode material of Mg-S batteries [54,86,87].

Furthermore, the study of electrolytes also accounts for a significant proportion of the research, and this is the focus of this review. 

## 3. Recent Developments in Electrolytes

The performance of Mg-S batteries is significantly influenced by the characteristics of the electrolyte used. In recent years, there has been an increasing focus on research pertaining to the electrolytes and mechanisms of Mg-S batteries (Figure 1b,c). This shift in focus is attributed to the realization among researchers that advancements in the electrode materials alone are insufficient to significantly enhance the electrochemical performance of Mg-S batteries. Only by elucidating the working mechanism of these batteries, addressing the shuttle effect, and enhancing the mass transfer efficiency can Mg-S batteries progress beyond the laboratory stage [88,89,90]. The ideal electrolyte for Mg-S batteries should facilitate the reversible deposition and stripping of magnesium at the anode and enable the efficient utilization of sulfur at the cathode [59,61,91]. Based on the chemical reactions, they can be classified into two categories: non-nucleophilic and nucleophilic electrolytes. A comparison of the key performance statistics of notable nucleophilic and non-nucleophilic electrolytes is shown at the end of Table 1.

### 3.1. Non-Nucleophilic Electrolytes

Owing to the electrophilic nature of elemental sulfur, it reacts with the nucleophilic substances in nucleophilic electrolytes, adversely affecting the stability and efficiency of batteries [52,73,74,77]. Therefore, in the design and application of Mg-S batteries and other sulfur-based batteries, researchers predominantly employ electrolytes with non-nucleophilic properties. In this section, there are two types of non-nucleophilic electrolyte systems in Mg-S batteries, which are chloride-containing and chloride-free.

#### 3.1.1. Chloride-Containing

**Figure 2:**
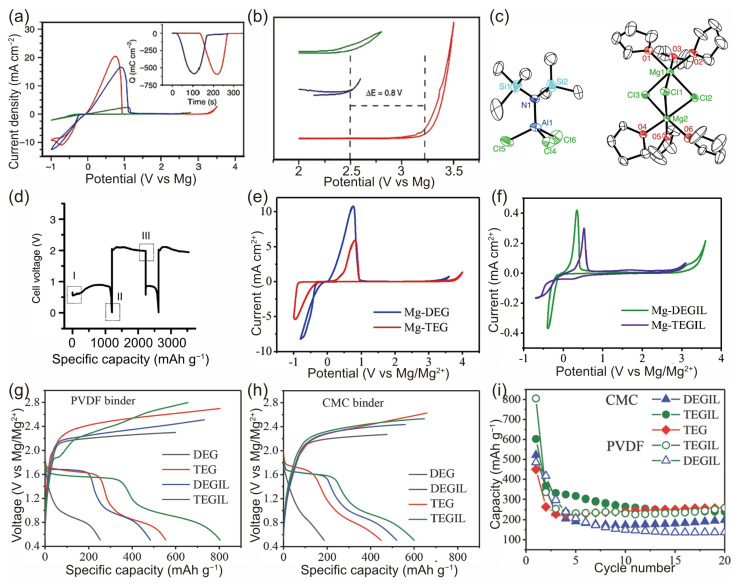
(**a**) Cyclic voltammograms of HMDSMgCl (green), the reaction product generated in situ from a 3:1 mixture of HMDSMgCl to AlCl_3_ (blue), and the crystal obtained from a 3:1 mixture of HMDSMgCl to AlCl_3_ (red). Inset shows the charge balance during the deposition and subsequent dissolution of Mg. (**b**) Enlargement of 2–3.5 V region of (**a**) highlighting the oxidative stability of the electrolytes. (**c**) ORTEP plot (25% thermal probability ellipsoids) of [Mg_2_Cl_3_·6THF][HMDSAlCl_3_]. Hydrogen atoms, THF crystallization, and second component of disorder are omitted for clarity. (**d**) Discharge and charge of a Mg-S coin cell at 50 and 25 μA, respectively. XPS spectra were taken from coin cells at various stages of cycling [52]. Cyclic voltammograms of the electrolyte in (**e**) diglyme solution (blue) and tetraglyme solution (red) and (**f**) diglyme/PP_14_TFSI solution (green) and tetraglyme/PP_14_TFSI solution (purple), using Pt as electrode at a scan rate of 25 mV s^−1^. Initial discharge–charge curves of S/CMK400PEG composite using (**g**) PVDF binder and (**h**) CMC binder in the electrolyte in diglyme (gray), tetraglyme (red), diglyme/PP_14_TFSI (blue), tetraglyme/PP_14_TFSI (green). (**i**) Cycling performance of S/CMK400PEG cathode in the electrolyte in diglyme/PP_14_TFSI (denoted as DEGIL in blue), in tetraglyme/PP_14_TFSI (denoted as TEGIL in green), and in tetraglyme (denoted as TEG in red) using CMC and PVDF as binders, respectively [55]. (**a**–**d**) Copyright © 2011, Hee Soo Kim et al. (**e**–**i**) © Copyright 2014 WILEY-VCH Verlag GmbH & Co. KGaA, Weinheim.

A moderate concentration of Cl^−^ is regarded as beneficial, not only for stabilizing Mg^2+^ but also for dissolving the passivating species on the Mg anode, thereby enhancing the Mg plating/stripping process. A hexamethyldisilazide magnesium chloride (HMDSMgCl)-based non-nucleophilic electrolyte, which exhibits good compatibility with sulfur cathodes, was synthesized in 2011 by H.S. Kim et al. [52]. The interaction between the HMDSMgCl electrolyte and the Lewis acid AlCl_3_ was investigated, with the objective of enhancing the electrochemical performance. By varying the acid-to-base ratio and reaction time, it was observed that the electrolyte’s electrochemical performance peaked after a 24-hour reaction period, specifically when the HMDSMgCl-to-AlCl_3_ ratio was 3:1. As illustrated by the green and blue lines in Figure 2a, the current density for Mg deposition exhibited an approximate sevenfold increase following the addition of AlCl_3_. However, the voltage stability of the HMDSMgCl electrolyte did not show any improvement (Figure 2b). To identify the reaction products of HMDSMgCl with AlCl_3_, crystals were harvested through the slow diffusion of hexane. The crystal structure [Mg_2_(μ-Cl)_3_·6THF][HMDSAlCl_3_] (Figure 2c) was determined using single-crystal X-ray diffraction. This structure was found to feature a cation with two octahedrally coordinated Mg centers, each containing three chlorine atoms. THF molecules, through oxygen coordination, occupy the remaining three sites on each Mg center. According to the constant current charging and discharging data, the specific capacity during the first discharge was approximately 1200 mAh g^−1^. However, the overpotential reached approximately 1.1 V, and the capacity rapidly decayed to around 395 mAh g^−1^ (Figure 2d). This study offers a novel perspective on the development of non-nucleophilic electrolytes for Mg-S batteries.

In order to synthesize the magnesium-bis-(hexamethyldisilazide) [(HMDS)_2_Mg] and AlCl_3_-based non-nucleophilic electrolyte in different ethers, a number of one-step strategies were employed by Z. Z. Karger et al. in 2013 [92]. Through the means of chemical processes involving magnesium bisamide and Lewis acids in aprotic solvents, the non-nucleophilic electrolyte for magnesium batteries was synthesized, which had an excellent electrochemical performance. The in situ process-generated electrolyte possessed excellent characteristics, such as a high anode stability, excellent ionic conductivity, good cycling efficiency, and feasibility of preparation. With these advantageous properties, it holds great promise in the area of rechargeable magnesium batteries.

To advance the performance of Mg-S batteries, Z. Z. Karger et al. explored a novel preparation method for non-nucleophilic electrolyte solutions using a two-step reaction in one pot [55]. This study initially employed (HMDS)_2_Mg-based diglyme and tetraglyme electrolyte solutions for constructing Mg-S batteries, integrating *N*-methyl-*N*-butylpiperidinium bis(trifluoromethanesulfonyl)imide (PP_14_TFSI) as an additive into these electrolytes. During the testing phase with a sulfur cathode (S/CMK), the electrolytes based on diglyme and tetraglyme demonstrated distinct capacities of 250 and 550 mAh g^−1^, respectively. However, the Mg-S batteries exhibited low-capacity retention, with a discharge potential of 1.65 V. Furthermore, the study also assessed the role of the ionic liquid (IL) PP_14_TFSI as a cosolvent in the electrolyte. Utilizing a PVDF binder and a tetraglyme/PP_14_TFSI solution, the batteries initially delivered approximately 800 mAh g^−1^ in the first cycle, but this value was significantly reduced to around 350 mAh g^−1^ in the subsequent cycle. Conversely, the batteries containing TEGIL (tetraglyme and PP_14_TFSI) with binders of either PVDF or CMC maintained a stable reversible capacity of about 260 mAh g^−1^ after over 20 cycles (Figure 2e–i). This study elucidated that the electrochemical conversion of magnesium and sulfur demonstrates a fundamentally unique battery chemistry compared to Li-S systems. It was observed that the significant hysteresis between the discharge and charge voltages during cell cycling contributes to the capacity degradation of the batteries. This type of electrolyte has garnered considerable interest among researchers, with scholars like Vinayan et al. [79], Yu et al. [82], and many other researchers also utilizing it to investigate Mg-S batteries.

**Figure 3:**
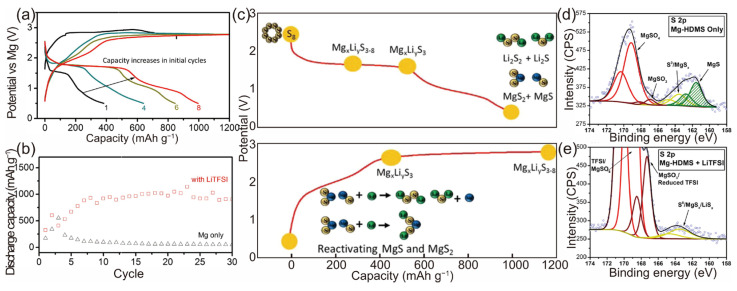
(**a**) Charge/discharge curves of sulfur cathode in 0.1 M Mg-HMDS + 1.0 M LiTFSI electrolyte in a three-electrode cell at a current of 71 mAh g^−1^ at room temperature. Arrow illustrates the capacity-increasing trend of the ACC/S composite cathode as a result of slow electrolyte penetration. (**b**) Cycling stability of the Mg-S battery in electrolyte with and without LiTFSI. (**c**) Working mechanism of the Mg-S battery with LiTFSI additive. Comparison of surface XPS measurements of Mg anode cycled in Mg-HDMS in the absence (**d**) and presence (**e**) of LiTFSI [17]. Copyright © 2015, American Chemical Society.

Previous research on lithium-ion and lithium–sulfur batteries has demonstrated that the physicochemical properties of electrolytes can be effectively enhanced with the addition of electrolyte additives. Furthermore, these additives can have a positive impact on the electrochemical performance of battery systems. In 2015, Gao et al. introduced a novel strategy to augment the reversibility of Mg-S chemistry [17]. A non-nucleophilic Mg electrolyte, supplemented with LiTFSI additives, facilitates the integration of a reversible polysulfide redox process into the cathode with Mg deposition/stripping at the anode. Galvanostatic charge–discharge tests revealed that the specific capacity of the primary magnesium electrolytes was approximately 650 mAh g^−1^. Notably, the electrolyte containing LiTFSI exhibited a reversible capacity of around 1000 mAh g^−1^ at 71 mA g^−1^, with a stable voltage plateau at 1.75 V, maintaining stability for 30 cycles (Figure 3a–e). This enhanced reversibility is attributed to two main factors: (1) Li^+^ ions participating in the cathode reaction, either forming readily rechargeable Li polysulfide (Li-PS) or integrating into the Mg-PS to create hybrid Mg/Li polysulfide (MgLi-PS) during discharge. (2) The hard Lewis acid characteristic of Li^+^ coordinating strongly with the surface S^2−^ of the lower-order Mg-PS, thereby increasing its solubility, reducing the reoxidation energy barrier, and rendering it electrochemically active. A reversible capacity of 1000 mAh g^−1^ is one of the highest shown in the Mg-S battery research so far, as shown in Table 1.

**Figure 4:**
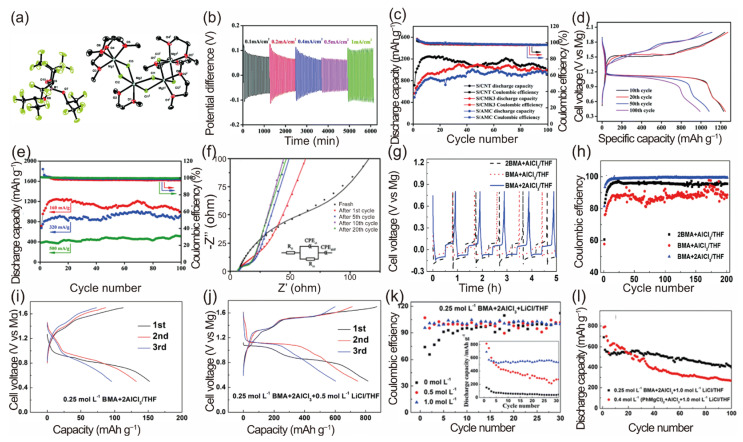
(**a**) ORTEP plot (50% thermal probability ellipsoids) of the molecular structure of crystalline [Mg_4_Cl_6_(DME)_6_][B(HFP)_4_]_2_. Hydrogen atoms are omitted for clarity. (**b**) Polarization properties of Mg/Mg symmetrical cells with the 0.5 M OMBB electrolyte at current densities of 0.1, 0.2, 0.4, 0.5, and 1 mA cm^−2^; the cycling time was 1 h per cycle (30 min charging and 30 min discharging). (**c**) Discharge capacities and Coulombic Efficiency as a function of the cycle number for different S-C composite cathodes at a current rate of 160 mA g^−1^ in the 0.5 M OMBB electrolyte. (**d**–**f**) Electrochemical characterization of the Mg-S-CNT battery in the 0.5 M OMBB electrolyte: (**d**) galvanostatic discharge–charge profiles for different cycles at a current rate of 160 mA g^−1^; (**e**) discharge capacities and Coulombic Efficiency at different charge–discharge current rates; (**f**) EIS measurements after different cycles (charge–discharge at a current rate of 160 mA g^−1^). The colored points represent the results of the tests, and the colored lines are the corresponding fitting curves [71]. The cycling curves (**g**) and cycling efficiency (**h**) of Mg plating/stripping on the SS substrate from 0.25 mol L^−1^ MBA–AlCl_3_/THF electrolytes with 2:1, 1:1, and 1:2 MBA-to-AlCl_3_ molar ratios. Discharge–charge profiles at 0.04C of the S@MC|Mg coin cell with 0.25 mol L^−1^ BMA + _2_AlCl_3_/THF electrolyte (**i**) and 0.25 mol L^−1^ BMA + 2AlCl_3_ + 0.5 mol L^−1^ LiCl/THF electrolyte (**j**). Cycling performance (inset) and Coulombic Efficiency at 0.04C of S@MC|Mg coin cells with 0.25 mol L^−1^ BMA + 2AlCl_3_/THF electrolytes containing LiCl at different concentrations (**k**). The cycling performance at 0.04 C of S@MC|Mg coin cells with 0.4 mol L^−1^ (PhMgCl)_2_ + AlCl_3_/THF and 0.25 mol L^−1^ BMA + 2AlCl_3_/THF electrolytes containing 1.0 mol L^−1^ LiCl (**l**) [93]. Copyright © The Royal Society of Chemistry 2017.

In 2017, Du et al. proposed an organic magnesium borate-based (OMBB) electrolyte, predominantly comprising a tetrakis(hexafluoroisopropyl) borate anion [B(HFP)_4_]^−^ (Figure 4a) and a solvating cation [Mg_4_Cl_6_(DME)_6_]^2+^ [71]. This electrolyte was synthesized using a simple in situ process involving tris(hexafluoroisopropyl)boronic acid [B(HFP)_3_], MgCl_2_, and Mg powder in 1,2-dimethoxyethane (DME). The overpotential was approximately 0.07 V at a current density of 0.1 mA cm^−2^ and increased marginally, with a rise in the current density from 0.1 mA cm^−2^ to 1 mA cm^−2^ (Figure 4b). Various sulfur–carbon composite materials (S-AMC, S-CNT, and S-CMK), prepared using the melt diffusion method, were employed to evaluate the OMBB electrolyte’s compatibility. Among these sulfur–carbon composite cathodes, tested at a current density of 160 mA g^−1^, the highest specific discharge capacity achieved was 1247 mAh g^−1^ (Figure 4c). Additionally, the S-CNT cathode exhibited a capacity retention rate of 80.4% after 100 cycles. The specific capacity of the sulfur–carbon composite cathodes increased initially, which may be attributed to the 0.5 M OMBB electrolyte’s self-conditioning effect, as it gradually permeated into the sulfur–carbon composite cathode. Both the discharge and charge curves feature two distinct voltage plateaus (Figure 4d). Remarkably, the Mg-S-CNT battery maintained a discharge capacity of about 500 mAh g^−1^ even at a current rate of 500 mA g^−1^. Electrochemical impedance spectroscopy (EIS) measurements (Figure 4f) were conducted to investigate the reasons for the initial increase in specific capacity. These measurements revealed a sharp decrease in the charge transfer resistance (R_ct_) during the initial cycles, elucidating the enhanced discharge capacity observed.

In 2019, Zhao et al. synthesized a remarkable magnesium electrolyte through the reaction of a magnesium salt, magnesium bis(diisopropyl)amide (MBA), and AlCl_3_ in THF [93]. For this Mg electrolyte, achieving a low overpotential and high Coulombic Efficiency during long-term cycling for Mg electrochemical plating/stripping is paramount. From the initial cycle, the overpotentials for Mg plating/stripping in the three electrolytes were consistently below −0.2 V and 0.1 V, respectively (Figure 4g). The corresponding Coulombic Efficiency (Figure 4h) is based on the ratio of the charge amount for magnesium plating to that of magnesium stripping. Notably, when the concentration of the MBA + 2AlCl_3_/THF electrolyte is 0.25 mol L^−1^, it exhibits the highest and most stable Coulombic Efficiency. The Coulombic Efficiency is, however, lower than other notable electrolytes, as demonstrated in Table 1.

To verify the compatibility of the electrolyte with the sulfur cathode, researchers assembled a coin cell using a 0.25 mol L^−1^ BMA + 2AlCl_3_/THF electrolyte, a S@MC cathode, and a Mg anode. This cell provided an initial discharge capacity of 152.0 mAh g^−1^ and a charge capacity of 112.8 mAh g^−1^, with a Coulombic Efficiency of 74.2%, (Figure 4i). The existing research indicates that incorporating lithium ions into magnesium electrolytes typically activates the electrochemistry of magnesium–sulfur battery systems. The first three galvanostatic discharge–charge curves of the S@MC|Mg coin cell with a 0.25 mol L^−1^ BMA + 2AlCl_3_ + 0.5 mol L^−1^ LiCl/THF electrolyte at 0.04C show an initial discharge capacity of about 815.6 mAh g^−1^ (Figure 4j). The capacities for the second and third cycles are approximately 747.5 mAh g^−1^ and 602.2 mAh g^−1^, respectively. The presence of Li^+^ has notably enhanced the reversibility of the sulfur cathode. Additionally, the inclusion of LiCl significantly improves the cycling stability (Figure 4k). The Coulombic Efficiency of S@MC|Mg coin cells with 0.25 mol L^−1^ BMA + 2AlCl_3_/THF electrolytes containing varying concentrations of LiCl are displayed in Figure 4k. The Coulombic Efficiency’s stability improves with an increasing LiCl concentration, especially at 1.0 mol L^−1^, where the charge capacity gradually aligns with, but does not exceed, the discharge capacity. These results suggest that the cathode’s reversibility is contingent on the presence of Li^+^. The enhanced interfacial compatibility and improved electrochemical performance may result from increased solution conductivity (1144 and 1185 μS cm^−1^ for 0.5 and 1.0 mol L^−1^ LiCl, respectively). The S@MC|Mg coin cell with a 0.25 mol L^−1^ BMA + 2AlCl_3_ + 1.0 mol L^−1^ LiCl/THF electrolyte demonstrates better cycling stability than one with a 0.4 (PhMgCl)_2_ + AlCl_3_ + 1.0 mol L^−1^ LiCl/THF electrolyte (Figure 4l). After 100 cycles, the capacity retention is approximately 57.7% and 34.4% of the initial capacity, respectively. However, this experiment could be considered controversial, as the Mg-S battery’s performance gains also correlate with the increasing concentration of lithium ions. It is important to consider what level of contribution the lithium ions are making to the performance and to question whether the battery can still be called a Mg-S battery and still comes with the associated benefits of a Mg-S battery compared to a Li^+^ battery. 

In 2019, Yang et al. introduced a novel electrolyte based on magnesium trifluoromethanesulfonate (Mg(CF_3_SO_3_)_2_)–AlCl_3_–MgCl_2_–anthracene–LiCl dissolved in THF and tetraglyme [53]. Mg(SO_3_CF_3_)_2_, which served as the source of the Mg^2+^ ions, is non-nucleophilic, easier to handle, and more cost-effective compared to Mg(TFSI)_2_ (where TFSI = bis(trifluoromethanesulfonyl)imide). However, challenges in Mg deposition/dissolution were observed, marked by a high overpotential, attributed to an inherent oxide layer on the Mg anode. In response, inspired by transmetalation reactions, where magnesium compounds react with Lewis acids, a typical Lewis acid, AlCl_3_ containing Cl^−^, was added. This addition reacted with Mg(CF_3_SO_3_)_2_ to generate effective active species within the solutions.

**Figure 5:**
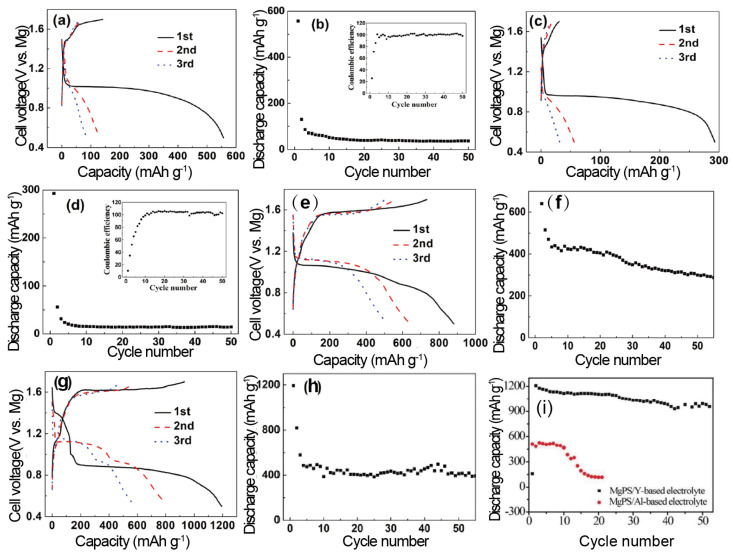
Discharge–charge curves and cycling performance of S@MC|Mg coin cell with 0.125 M Mg(CF_3_SO_3_)_2_ + 0.25 M AlCl_3_ + 0.25 M MgCl_2_ + 0.025 M anthracene/THF + TG (1:1 volume ratio) electrolyte (**a**,**b**) and 0.25 M Mg(CF_3_SO_3_)_2_ + 0.5 M AlCl_3_ + 0.25 M MgCl_2_ + 0.025 M anthracene/THF + TG (1:1 volume ratio) electrolyte (**c**,**d**) between 0.5 and 1.7 V at 0.05 C. Discharge–charge profiles and cycling performance of S@MC|Mg coin cell with 0.125 M Mg(CF_3_SO_3_)_2_ + 0.25 M AlCl_3_ + 0.25 M MgCl_2_ + 0.025 M anthracene/THF + TG (1:1 volume ratio) electrolyte adding 0.5 M LiCl (**e**,**f**) or LiCF_3_SO_3_ (**g**,**h**) between 0.5 and 1.7 V at 0.05 C [53]. (**i**) Cycling stability of the MgPS/Y-based electrolyte and MgPS/Al-based electrolyte cells under a current density of 80 mA g^−1^ [95]. Copyright © 2019, American Chemical Society.

A Mg-S@microporous carbon cell, incorporating a 0.125 M Mg(CF_3_SO_3_)_2_ + 0.25 M AlCl_3_ + 0.25 M MgCl_2_ + 0.025 M anthracene/THF and tetraglyme (1:1, *v*/*v*) electrolyte, was cycled at 0.05 C (Figure 5a,b). The Coulombic Efficiency of the cell initially reached almost 100% after a few cycles. However, due to substantial unreacted sulfur and inefficient Mg ion dissociation within the cathode, the discharge capacity sharply declined to just 50 mAh g_sulfur_^−1^ after 50 cycles.

The previous research indicates that the issue of polysulfide shuttling in electrolytes can be mitigated by increasing the electrolyte concentration. In such high-concentration electrolytes, fewer sulfur molecules, either as elemental sulfur or polysulfides, dissolve into the electrolyte during cycling. Consequently, this approach helps in curtailing the loss of active material. Furthermore, the discharge–charge performance of a S@MC|Mg coin cell containing a concentrated electrolyte (0.25 M Mg(CF_3_SO_3_)_2_ + 0.5 M AlCl_3_ + 0.25 M MgCl_2_ + 0.025 M anthracene/THF + TG (1:1 volume ratio)) was evaluated. This configuration not only led to a significant reduction in the specific capacity of the coin cell but also resulted in a poorer cycle performance compared to using a dilute electrolyte (Figure 5c). Notably, the Coulombic Efficiency exceeded 100% during stable cycling (Figure 5d).

The incorporation of Li^+^ ions into the electrolyte, with the aim of facilitating the dissolution of Mg^2+^ ions and thereby reducing the kinetic barriers while increasing the solubility of low-order polysulfides, was undertaken to enhance the electrochemical performance. The researchers introduced LiCl (Figure 5e,f) and LiCF_3_SO_3_ (Figure 5g,h), respectively, into a 0.125 M Mg(CF_3_SO_3_)_2_ + 0.25 M AlCl_3_ + 0.25 M MgCl_2_ + 0.025 M anthracene/THF and tetraglyme (1:1, *v*/*v*) solution. The results (Figure 5e,f) demonstrate that the addition of LiCl prolonged the discharge plateau at 1.05 V, leading to an enhanced discharge capacity and reversibility, confirming that Li^+^ ions effectively promote Mg dissolution. Notably, adding 0.5 M LiCF_3_SO_3_ as an additive further improved the cell performance. The cell achieved a discharge capacity of approximately 400 mAh g_sulfur_^−1^ at 0.05 C over 50 cycles, suggesting that the addition of CF_3_SO_3_Li also mitigates the detrimental effects on Mg plating. However, this method also faces the same controversy mentioned before: can the battery still be classified as a Mg-S battery, which maintains a high level of safety and is less prone to dendrites than one with the addition of Li^+^? In recent years, the majority of researchers have encountered this problem and have stopped adding Li^+^ into Mg-S batteries. 

It is significant to note that in 2014, Zhao-Karger et al. reported in their study the effect of augmenting the electrolyte viscosity by incorporating high-viscosity PP_14_TFSI (173 mPa s), which can partially impede the movement of polysulfides toward the anode [55]. This method demonstrates a novel strategy for mitigating the shuttle effect in Mg-S batteries. By enhancing the electrolyte’s viscosity, the mobility of the polysulfides is decreased, thus curtailing their transference and subsequently reducing the adverse impacts on the battery performance.

It is noteworthy that in 2021, Sun et al. described reduced perylene diimide-ethylenediamine (rPDI) as an efficacious electrolyte additive. They added 0.2 mM rPDI into a Mg(TFSI)_2_–MgCl_2_-based electrolyte, which adsorbed onto the Mg and repelled the TFSI^−^ anions away from the Mg surface, preventing TFSI^−^ decomposition and Mg passivation [94]. The full cell showed a highly stable cycle life at 15 C (>1000 cycles), revealing the mitigation of the shuttle effect. Although the capacity is low at 110 mAh g^−1^ after 1000 cycles, it is a significant improvement over other electrolyte systems, as can be seen in Table 1, where it has the largest number of cycles reported.

In recent years, there have been significant advancements in the field of novel Lewis acids. In 2018, Xu et al. developed an yttrium (Y)-based electrolyte by replacing AlCl_3_ with YCl_3_ [95]. Their findings revealed several key advantages: firstly, the standard electrode potential of Y ions (−2.372 V vs. SHE) is higher than that of Al ions (−1.66 V vs. SHE), and secondly, YCl_3_ effectively facilitates the removal of water from the electrolyte. In their synthesis, MgCl_2_ (1 molar equivalent) and YCl_3_ (2 molar equivalent) were reacted with diglyme in an ionic liquid solvent, PYR14TFSI, at a temperature of 120 °C.

To investigate the distinct impacts of the YCl_3_ and AlCl_3_ additives, Xu et al. compared the electrochemical performances of two Mg-S cells: one with a MgPS cathode in an aluminum-based electrolyte and the other with a MgPS cathode in an yttrium-based electrolyte. The cell featuring the yttrium-type electrolyte demonstrated stable cycling for 50 cycles with a discharge capacity of approximately 900 mAh g^−1^ (Figure 5i). In contrast, the cell containing the aluminum-based electrolyte only managed 20 cycles before experiencing a sharp decline in capacity. Furthermore, the electrochemical impedance spectroscopy (EIS) data indicated that the yttrium-based electrolyte exhibited a lower impedance [95].

The inclusion of chloride ions has been shown to provide a multitude of benefits, such as stabilization of the Mg^2+^ ions and amplification of the dissolution of passivating substances on the Mg anode. This subsequently facilitated improved efficiency in the plating and stripping of Mg. The impact is particularly pronounced when employing HMDSMgCl-based electrolytes, where the introduction of AlCl_3_ results in a significant rise in current density for Mg deposition, although it does not enhance the voltage stability. Additionally, the advancement of organic magnesium borate-based (OMBB) electrolytes and the exploration of innovative techniques for preparing non-nucleophilic electrolyte solutions have further elevated the performance of Mg-S batteries.

#### 3.1.2. Chloride-Free

**Figure 6:**
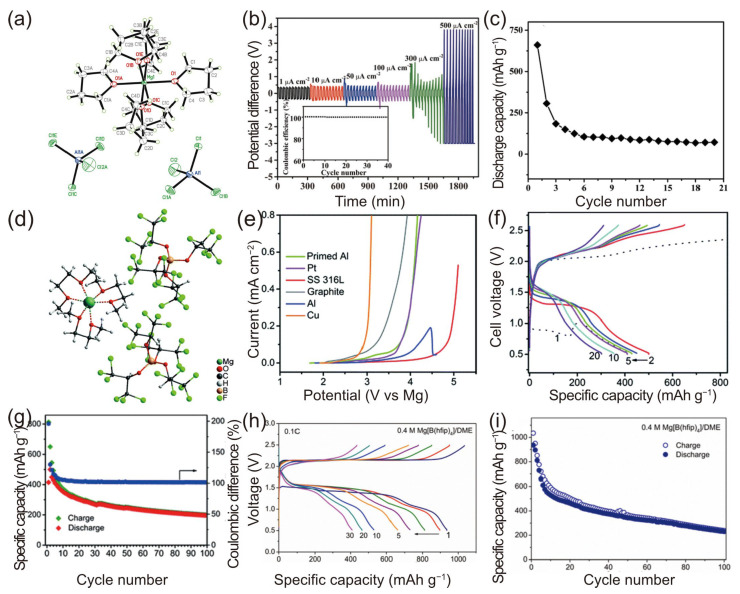
(**a**) Proposed formation mechanism and X-ray crystal structure of Mg salt. (**b**) Cycling behavior of a symmetrical cell with electrolyte [Mg(THF)_6_][AlCl_4_]_2_ in PYR14TFSI/THF (1:1 *v*/*v*) at different current densities of 1 μA cm^−2^ to 500 μA cm^−2^; the cycle time was 30 min per cycle (15 min charging and 15 min discharging). (**c**) Discharge and charge profile for 20 cycles of discharging and charging, cathode: 50 % S loading, NG/SP/commercial S/PVDF = 4:5:10:1. Rate: 0.01 C discharging, 0.02 C charging. Anode: Mg disk [77]. (**d**) Ball-and-stick representation of the Mg[B(hfip)_4_]_2_·3DME crystal. (**e**) Linear sweep voltammograms of various electrodes at a scan rate of 5 mV s^−1^. Battery performance of the Mg-S-CMK-3 cell: (**f**) discharge–charge profiles, (**g**) cycling behavior [51]. (**h**) Charge/discharge profiles; (**i**) cycling performance of the ACCS–Mg cell with 0.4 M electrolytes [84]. (**a**–**c**) *©* 2014 WILEY-VCH Verlag GmbH & Co. KGaA, Weinheim. (**d**–**g**) Copyright © The Royal Society of Chemistry 2017. (**h**,**i**) Copyright © 2019, American Chemical Society.

Muldoon et al. highlighted the adverse effects associated with the presence of chlorine in electrolytes, pointing out that the chlorides in the electroactive species [Mg_2_(µ-Cl)_3_·6THF] are a primary cause of corrosion [97]. Additionally, the bulky structure of the cation, characterized by two octahedrally coordinated Mg atoms linked by three chlorides, impedes Mg ion mobility. This analysis underscores the urgent need to develop and synthesize new types of chlorine-free salts for use in Mg-S batteries. In 2016, Li et al. pioneered a method for synthesizing a simple chloride-free [Mg(THF)_6_]^2+^ cation salt with AlCl^4−^ as a counteranion, utilizing a straightforward heating process in an ionic liquid solvent [77]. The crystal structure of the [Mg(THF)_6_][AlCl_4_]_2_ salt is illustrated in Figure 6a. Li et al. underscored the benefits of using ionic liquids, including their high boiling point, low melting point, exceptional chemical and thermal stability, nonflammability, and low vapor pressure [77,98]. They proposed a stoichiometric reaction of MgCl_2_ (1 molar equivalent) with AlCl_3_ (2 molar equivalents), using the ionic liquid n-methyl-(n-butyl) pyrrolidinium bis(trifluoromethanesulfonyl)imide (PYR14TFSI) as a reaction medium. It was postulated that the chloride ions in MgCl_2_ could be completely displaced by AlCl_3_ under elevated temperatures.

Initially, a magnesium stripping/plating experiment was conducted, with the current density varying from 1 μA cm^−2^ to 500 μA cm^−2^ and the cycling time for each charge and discharge set at 15 min. At lower current densities, the Coulombic Efficiency of the cell is nearly 100% (Figure 6b). However, as the current density escalates to 300 μA cm^−2^, an increase in polarization occurs, leading to irregular potential fluctuations. For further analysis, electrochemical impedance spectroscopy (EIS) was employed to examine the formation and stability of the interface between the electrolyte solution and the magnesium electrode over the course of the cycling. Initially, the interface between the magnesium electrode and the electrolyte solution is unstable, but it stabilizes after a certain number of cycles, evidenced by the gradual stabilization of the solid electrolyte interface (SEI) layer on the magnesium electrode. In the cycling performance of the Mg-S cells (Figure 6c), it was observed that the cell maintains stability for over 20 cycles, although its capacity decreases sharply in the first five cycles from approximately 700 mAh g^−1^ to 130 mAh g^−1^, before stabilizing at a relatively steady capacity of around 70 mAh g^−1^.

In 2017, Zhao-Karger et al. developed a fluorinated magnesium alkoxyborate-based electrolyte for Mg-S batteries [51]. They synthesized conductive salts via the reaction of Mg[BH_4_]_2_ with fluorinated alcohols (RF-OH) in ethereal solvents, such as DME. Upon removing the solvent from Hexafluoro-2-propanol (hfip), the conductive salt Mg[B(hfip)_4_]_2_·3DME was obtained. Its crystal structure, determined using X-ray crystallography, is Mg[B(hfip)_4_]_2_·3DME (Figure 6d). The crystal unit consists of typical ion pairs, with the octahedral coordination geometry around the Mg^2+^ ions being slightly deformed due to the solvation by the three DME molecules. In the [B(hfip)_4_]^−^ counteranion, the boron (B) atom is tetrahedrally bonded to four hexafluoroisopropyloxy groups. The O–B–O angles (107.8° and 107.2°) approximate the ideal tetrahedral angle. The anodic stability of the MgBOR(hfip)/DME electrolyte on conventional electrode substrates, including stainless steel (SS), Al, primed Al, and Cu, was examined using linear sweep voltammetry (LSV). The voltammogram indicates that the oxidation stability of the electrolyte on Pt is about 3.5 V, possibly limited by the DME oxidation (Figure 6e). Swagelok-type cells, containing a MgBOR(hfip)/DEG–TEG electrolyte, S/CMK-3 cathode, and Mg foil anode, were cycled at room temperature at 0.1 C (167 mA cm^−2^). The galvanostatic discharge/charge curves from the 1st to the 5th, 10th, and 20th cycles are displayed in Figure 6f. After the initial cycles, the discharge voltage stabilized at around 1.5V. From the second cycle onward, the discharge capacity decreased but remained above approximately 200 mAh g^−1^ sulfur up to the 100th cycle (Figure 6g). The overcharging behavior, evident in the first five cycles, led to a reduced efficiency. To optimize the electrochemical performance, the concentration of Mg[B(hfip)_4_]_2_ in the DME solvent varied from 0.1 to 0.4 M. Cyclic voltammetry revealed that higher concentrations of Mg[B(hfip)_4_]_2_ increased the current densities [84]. Based on these findings, the authors concluded that fluorinated alkoxyborate-based electrolytes hold promise for Mg-S batteries.

The same research team also explored Swagelok-type cells consisting of a 0.4 M MgBhfip/DME electrolyte, an ACCS cathode, and a Mg foil anode. These cells were cycled at a current rate of 0.1 C (167 mA cm^−2^) at 25 °C [84]. The initial galvanostatic discharge/charge curves displayed a relatively flat discharge voltage plateau at approximately 1.5 V, followed by a sloped region until a cutoff voltage of 0.5 V, suggesting a stepwise reaction pathway. The cells achieved a discharge capacity of about 930 mAh g^−1^, while the charge capacity marginally surpassed the discharge capacity (Figure 6h,i). The Coulombic Efficiency for the first cycle, calculated by dividing the charge capacity by the discharge capacity, was approximately 110%. This gradual decline in capacity is attributed to the dissolution of the magnesium polysulfide (MgSx) and the ongoing loss of active material.

**Figure 7:**
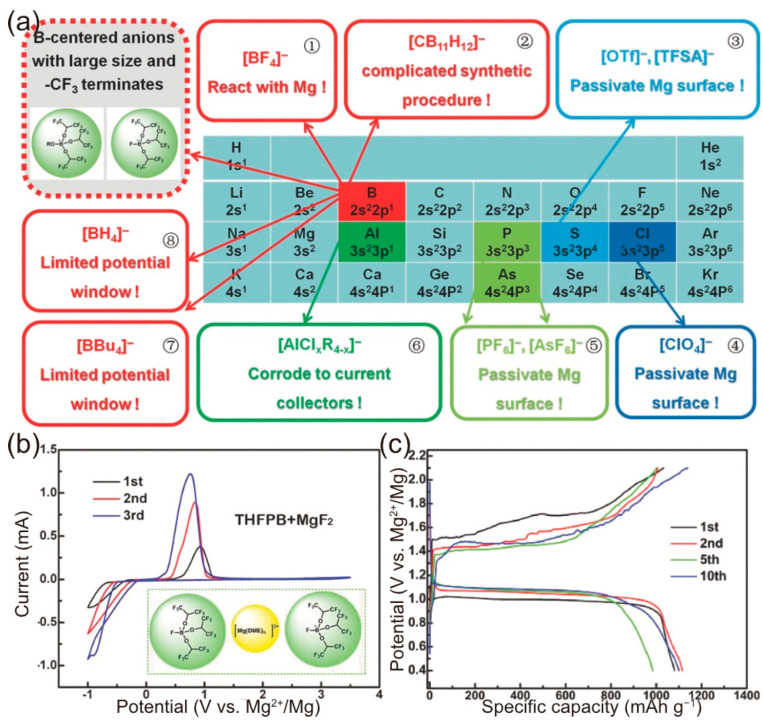
(**a**) Decorated periodic table for directing efficient Mg-ion electrolytes. Electrolytes derived from anions of ① to ⑧ suffer from specific problems. Electrolytes from ①, ③, ④, and ⑤ can react with Mg metals, forming impervious surface layers. Anions of ⑦ and ⑧ display insufficient anodic stability, resulting in limited potential windows of the determined Mg-ion electrolytes. Electrolytes containing anions of ⑥ corrode typical current collectors and show incompatibility with sulfur and oxide cathodes. The complicated synthetic procedure and incompatibility with sulfur cathodes of ②-constructed Mg-ion electrolyte makes it less promising for practical Mg-ion electrolytes. (**b**) CV curves of SS electrode in BCM electrolyte containing THFPB and MgF_2_ at 5 mV s^−1^. (**c**) The electrochemical performances of the Se/C and S/C electrodes in BCM electrolytes: galvanostatic charge/discharge profile [50]. Copyright © 2017 WILEY-VCH Verlag GmbH & Co. KGaA, Weinheim.

In 2017, Zhang et al. introduced a boron-centered anion-based magnesium electrolyte (BCM electrolyte) characterized by its ease of synthesis, high ionic conductivity, broad potential window (3.5 V vs. Mg), compatibility with electrophilic sulfur, and non-corrosiveness toward the cell components [50]. They analyzed the chemical and electrochemical properties of various anionic forms in non-nucleophilic electrolytes, detailing the specific challenges encountered in their practical applications, as highlighted in the colored boxes of Figure 7a. In their approach, the properties of the BCM electrolytes, including the electrochemical window, salt concentration, and compatibility with the Mg anode, could be finely tuned by selecting specific anionic groups. Notably, the use of tris(2*H*-hexafluoroisopropyl) borate (THFPB)/MgF_2_ salts in the DME solvent led to the formation of the [Mg(DME)_n_][FTHB]_2_ complex (Figure 7b), which demonstrated a wide operating window of 3.5 V. With a magnesium anode and a sulfur cathode containing 85 wt.% sulfur and a sulfur loading of 1.5 mg cm^−2^, the cell delivered a discharge capacity of 1081 mAh g^−1^ and a stable voltage plateau at 1.1 V and exhibited no overcharging in subsequent cycles (Figure 7c). The excellent capacity retention over 30 cycles, with an initial discharge capacity of 86.4%, underscores the success of this novel electrolyte design concept.

In 2021, Ren et al. demonstrated for the first time the use of a PP_14_TFSI ionic liquid as a co-solvent with THF in a chlorine-free MBA-based electrolyte [60]. This approach significantly enhanced the ionic conductivity and increased the oxidative stability potential on stainless steel to 2.2 V vs. Mg/Mg^2+^. The experimental results indicated that this electrolyte exhibited a low overpotential below 200 mV and maintained approximately 90% Coulombic Efficiency in the reversible electrochemical plating/stripping of magnesium. Following the addition of PP_14_TFSI, the current density for magnesium plating/stripping was escalated by a factor of 238. Additionally, the MBA-2AlF_3_ electrolyte showed good compatibility with the Mo_6_S_8_ cathode. Furthermore, Ren et al. also reported the performance of a Se@pPAN|Mg full cell, which delivered an initial capacity of 447.8 mAh g^−1^ at 0.2 C, with a minimal capacity decay of about 0.66% per cycle over more than 70 cycles.

The chloride-free electrolytes highlighted in this section, such as the fluorinated alkoxyborate-based electrolytes and boron-centered anion-based (BCM) electrolytes, demonstrate wide operating windows, high ionic conductivities, and good compatibility with Mg anodes and sulfur cathodes. Chloride-free electrolytes mitigate the disadvantages shown in chloride-containing electrolytes of corrosion and limited Mg ion mobility. The synthesis of simple cation salts, such as [Mg(THF)_6_]^2+^ with non-chloride counterions, and the development of fluorinated magnesium alkoxyborate-based electrolytes signify great strides toward the goal of achieving high-performance Mg-S batteries. These advancements are not merely academic; they offer practical pathways to enhance the electrochemical performance, stability, and safety of Mg-S batteries.

### 3.2. Nucleophilic Electrolytes

The reaction between PhMgCl and AlCl_3_ facilitates the synthesis of Grignard reagent-based nucleophilic all-phenyl complex (APC) electrolytes. Initially, these APC electrolytes were not tailored to use in Mg-S batteries due to the high nucleophilicity of organomagnesium compounds, which generally renders them incompatible with sulfur-based conversion cathodes. However, in 2017, Linqi Zeng et al. adapted an APC electrolyte for Mg-S battery applications [96]. Utilizing copper as the current collector for a sulfur cathode and integrating it with the nucleophilic (PhMgCl)_2_-AlCl_3_/THF electrolyte, they achieved an initial discharge capacity of 659 mAh g^−1^. Additionally, at a current density of 10 mA g^−1^, the reversible capacity of the cell stabilized at 113 mAh g^−1^ after 20 cycles.

**Figure 8:**
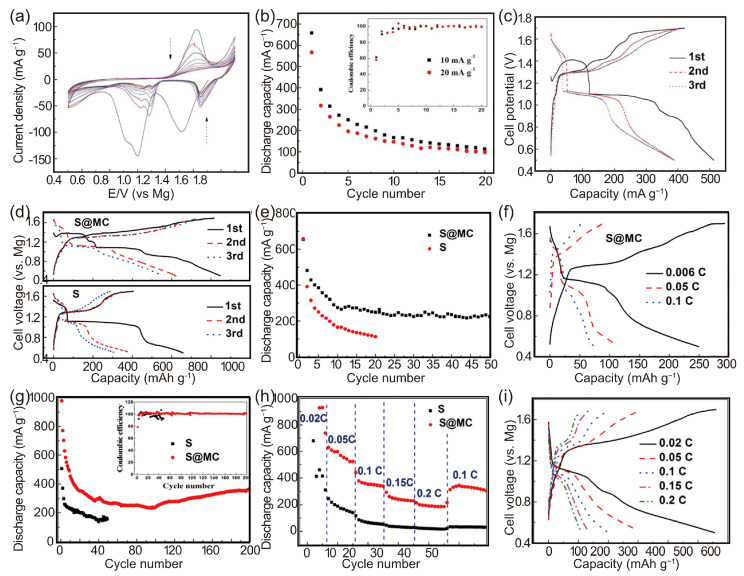
(**a**) CVs of the Mg-S coin cell with Cu as the cathode current collector at 0.05 mV s^−1^. (**b**) The cycling performance of Mg-S coin cells with Cu as the cathode current collector at 10 mA g^−1^ and 20 mA g^−1^. (**c**) Initial three discharge–charge curves of Mg-S cell with Cu as the cathode current collector in 0.4 mol L^−1^ (PhMgCl)_2_-AlCl_3_ + 1.0 mol L^−1^ LiCl/THF electrolyte at 10 mA g^−1^ [96]. Initial three discharge–charge curves (**d**) and cycling performance (**e**) of elemental sulfur and S@MC at a rate of 0.006 C. (**f**) The 20th discharge–charge curves of S@MC at different rates; the electrolyte is 0.4 mol L^−1^ (PhMgCl)_2_–AlCl_3_/THF. (**g**) Cycling performance of elemental sulfur and S@MC composite at 0.1 C; inset is the Coulombic Efficiency upon cycling. (**h**) The rate performance of elemental sulfur and S@MC composite at different rates. (**i**) Typical discharge–charge curves of S@MC at different rates; the electrolyte is 0.4 mol L^−1^ (PhMgCl)_2_–AlCl_3_ + 1.0 mol L^−1^ LiCl/THF [54]. (**a**–**c**) © Linqi et al., 2017. Published by ECS. (**d**–**i**) Copyright © 2018, American Chemical Society.

The cyclic voltammetry (CV) curves of the Mg-S coin cell at a scan rate of 0.05 mV s^−1^ (Figure 8a) feature Cu as the cathode current collector. During the first cathodic scan, two significant reduction peaks at 1.0 V and 1.35 V were observed, accompanied by shoulder peaks at 0.9 V. These peaks are indicative of the formation of higher-order magnesium polysulfides (MgSx, where 4 ≤ X ≤ 8). These high-order polysulfides undergo reduction into their lower-order counterparts, subsequently leading to the formation of MgS_2_ and MgS. In the anodic scan, the re-oxidation of MgS and MgS_2_ back into polysulfide MgSx (x > 2) is characterized by a distinct oxidation peak at 1.43 V. They observed an increase in current above 1.6 V, which suggests further oxidation into higher-order polysulfides or elemental sulfur. A marked decrease in the peak currents during the second cathodic scan points to the dissolution of some of the sulfur or polysulfides into the electrolyte, contributing to a decline in cell capacity. The shift in the peak voltage aligns with these electrochemical processes.

The discharge–charge cycling performance and corresponding Coulombic Efficiency of a Mg-S coin cell with a Cu cathode collector operating at a current density of 10 mA g^−1^ are displayed in Figure 8b. The cell’s capacity exhibited a steady decline until the 20th cycle, stabilizing at approximately 113 mAh g^−1^ thereafter. Notably, after a few initial cycles, there was a gradual increase in the charging capacity compared to the discharge capacity. The Coulombic Efficiency, exceeding 100% (Figure 8b), is attributed to the polysulfide shuttle effect, a recognized parasitic side reaction. Furthermore, elevating the current density to 20 mA g^−1^ proved effective in maintaining the discharge capacity above 99 mAh g^−1^ after 20 cycles, indicating a mitigation of the dissolution and shuttle effects. This improvement suggests a robust interaction between Cu and S, along with the enhanced electronic conductivity of copper sulfides, positively affecting the sulfur utilization and the cyclic stability of the sulfur cathode in the nucleophilic electrolyte. Additionally, incorporating LiCl into the (PhMgCl)_2_-AlCl_3_/THF electrolyte further enhanced its cycling stability and rate performance. The capacity initially decreased from 512 mAh g^−1^ to 388 mAh g^−1^ and then was consistently held at 384 mAh g^−1^ (Figure 8c).

In 2018, expanding on their previous work, W. Wang et al. from the same research group introduced an innovative sulfur@microporous carbon (S@MC) electrode [54]. This electrode, employing copper as the current collector, was designed as a novel cathode for advanced Mg-S batteries. It effectively utilizes APC-based nucleophilic electrolytes, marking a significant advancement in the development of high-performance Mg-S battery technology.

Microporous carbon (MC), serving as the host material, enhances the chemical kinetics of the electrode and adsorbs sulfur and polysulfides effectively. At 50 °C, the formation of copper sulfide occurs when sulfur is coated onto a Cu collector, creating a robust chemical interaction between the Cu and sulfur. This interaction plays a crucial role in preventing the sulfur from being eroded by the electrolyte and reducing the rate of polysulfide dissolution. Utilizing the unique properties of microporous carbon and Cu current collectors, the initial discharge capacity of the S@MC electrode is around 979.0 mAh g^−1^. After undergoing 200 cycles at a 0.1 C rate, the capacity reaches a stable value of 368.8 mAh g^−1^, indicating improved sulfur utilization and enhanced cycle stability. This retention rate is on par with several non-nucleophilic electrolytes [52,79]. Even at a higher rate of 0.2 C, the composite maintains a capacity of about 200 mAh g^−1^, achieving a Coulombic Efficiency of up to 200%. The strategy of reinforcing the chemical bonds between smaller S_2-4_ molecules and larger S_8_ molecules within the MC framework has been demonstrated to be an effective approach to boosting the cyclic stability, rate performance, and sulfur utilization in Mg-S batteries with nucleophilic electrolytes (Figure 8d–i).

**Figure 9:**
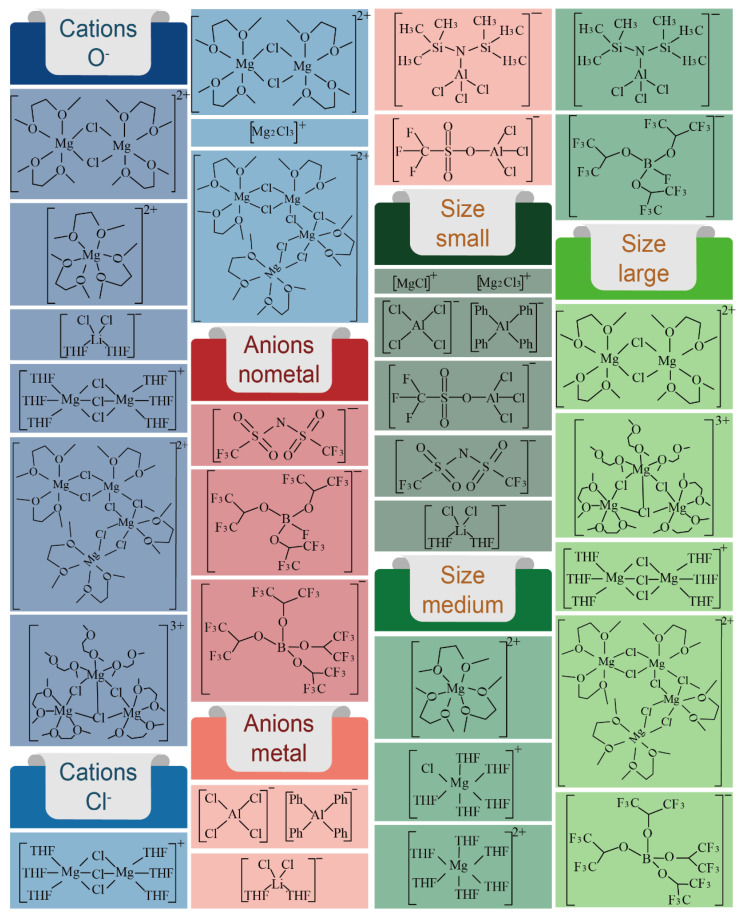
All clearly identified electroactive species in the electrolytes investigated so far.

Research on electrolyte systems suitable for Mg-S batteries is still ongoing; nonetheless, some achievements have already been accomplished. Figure 9 summarizes all the clearly identified electroactive species in the electrolytes investigated so far. According to the structure of these electroactive species, we have divided them into cations: O^−^, cations: Cl^−^, anions: non-metal, anions: metal, size: small, size: medium, and size: large. 

The electrolyte plays a pivotal role in the performance of Mg-S batteries, directly influencing their efficiency, capacity, cycling stability, and safety. The choice between non-nucleophilic and nucleophilic electrolytes remains unclear. The addition of chloride ions then adds a further layer of complexity. Finding the ideal electrolyte that balances conductivity, reactivity, and stability with the Mg anode and sulfur cathode is still a challenging goal.

Non-nucleophilic electrolytes have shown promise due to their ability to stabilize Mg^2+^ ions and facilitate efficient plating/stripping processes. The shuttle effect and the need for a higher Coulombic Efficiency remain as the biggest issues. Chloride-free electrolytes address the corrosion issues associated with chloride ions but require careful synthesis to maintain high ionic conductivity and compatibility with sulfur cathodes. Nucleophilic electrolytes offer unique pathways for Mg-S battery configurations, but the reactivity of organic magnesium compounds with sulfur-based cathodes needs to be addressed. The use of copper current collectors and microporous carbon in the literature has shown that there is great promise for the future of these electrolytes.

This review has analyzed a number of notable electrolytes which have been used in the field of Mg-S battery research. Table 1 provides a comparison of these electrolytes, highlighting key statistics such as Coulombic Efficiency, capacity, and current rate. It clearly reveals a major difficulty that magnesium–sulfur batteries are currently facing with low cycling performances. This is mainly due to the sharp drop in the battery capacity caused by the shuttle effect.

## 4. The Mechanism of Sulfur Reduction

In 2012, Muldoon et al. emphasized the necessity of using non-nucleophilic magnesium organohaloaluminate electrolytes to achieve an effective pairing between magnesium and sulfur [48]. In 2014, Zhirong et al. used modified electrolytes in tetraglyme or a binary solvent of glyme and PP_14_TFSI. The use of this additive aims to adjust the viscosity of the electrolyte and may reduce the solubility of magnesium polysulfides [55]. The result shows the ability of the glyme polyether chains to permit multidentate cation coordination through the oxygen atoms, allowing for the adoption of flexible coordination numbers and geometries, which considerably improves the performance, Coulombic Efficiency, and discharge voltage. Then, they propose a redox mechanism for Mg-S batteries:

Step I: Elemental sulfur is reduced into MgS_8_ at the solid electrolyte interface (SEI), followed by the dissolution of MgS_8_ into a liquid cathode, transitioning into lower-order polysulfides.

(1) $$Step\;I\;S_{8}+4e^{-}+{Mg}^{2+}\to 2MgS_{4}$$

Step II: Low-order polysulfides such as MgS_4_ are reduced into MgS_2_, corresponding to the second discharge platform.

(2) $$Step\;II\;MgS_{4}+2e^{-}+{Mg}^{2+}\to 2MgS_{2}$$

Step III: MgS_2_ is reduced into MgS, a process characterized by high kinetic barriers and polarization.

(3) $$Step\;III\;MgS_{2}+2e^{-}\to 2MgS$$

In the work of Robba et al., 2017, they used a non-nucleophilic electrolyte solution prepared from MgCl_2_ and Mg(TFSI)_2_ salts dissolved in a binary mixture of ether solvents [67]. The results show the battery exhibited two clear discharge plateaus during the first discharge process, corresponding to the conversion of MgS_x_ (high-voltage plateau) and the further conversion of polysulfides into MgS (low-voltage plateau). In the same year, Gao et al. determined the specific discharge plateau in a MgTFSI_2_–DME electrolyte:

Step I: The transformation of elemental sulfur into long-chain polysulfides (2.4–1.5 V potential slope)

(4) $$Step\;I\;S_{8}+4e^{-}+{Mg}^{2+}\to MgS_{8}\;2.5–1.5\;V$$

Step II: Shortening of the polysulfide chain (1.5 V potential plateau)

(5) $$Step\;II\;MgS_{8}+6e^{-}+3{Mg}^{2+}\to 4MgS_{2}\;1.5\;V$$

Step III: The solid-state transition from short-chain polysulfides to magnesium sulfide (1.5–0.5 V potential slope)

(6) $$Step\;III\;MgS_{2}+2e^{-}\to 2MgS\;1.5–0.5\;V$$

**Figure 10:**
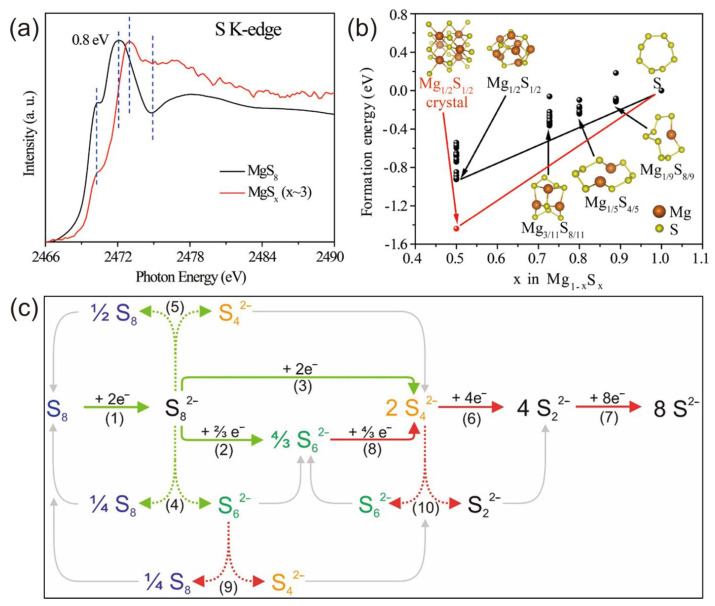
(**a**) The S K-edge XAS spectra of the MgS_8_ and MgS_x_ (x∼3) reference samples. (**b**) Calculated structures of magnesium polysulfides and their formation energies [68]. (**c**) Proposed reduction pathway for sulfur species in glyme-based electrolytes [99]. (**a**,**b**) Copyright © 2019, American Chemical Society. (**c**) Copyright © 2021 Joachim et al. Published by American Chemical Society.

In 2019, Yan et al. further investigated the sulfur reduction reaction route according to the in situ method using an electrolyte of Mg(HMDS)_2_–AlCl_3_ [68], also including three stages (Figure 10b):

Stage I: Rapid reaction stage for the formation of high-order MgS_x_ (MgS_8_, MgS_4_).

(7) $$Stage\;I\;S_{8}+{Mg}^{2+}+e^{-}\to MgS_{8}+MgS_{4}$$

Stage II: Reduction stage from high-order MgS_4_ into Mg_3_S_8_

(8) $$Stage\;II\;MgS_{8}+MgS_{4}+e^{-}\to {Mg}_{3}S_{8}$$

Stage III: Sluggish further reduction of Mg_3_S_8_ into MgS; this reaction predominantly occurs in the solid phase, contributing to the rapid capacity decay of the Mg-S battery.

(9) $$Stage\;III\;{Mg}_{3}S_{8}+e^{-}\to MgS$$

In situ XAS analysis (Figure 10a) reveals that Mg_3_S_8_ and MgS are electrochemically inert and difficult to convert back into high-order polysulfides. This leads to a rapid decline in the battery capacity and a shortened cycle life.

Forrest et al. investigated the impact of dissolved sulfur on the passivation of Mg anodes in Mg-S batteries, revealing that interactions between the dissolved polysulfides and the Mg anode led to the formation of a passivating MgS layer on the anode surface [25]. This layer readily reforms during reduction but can be removed under oxidative conditions. The research highlights that the concentration of dissolved S_8_ influences the rate of MgS layer formation by altering the equilibrium of polysulfide disproportionation.

In 2022, Joachim et al. characterized the magnesium polysulfide dissolution behavior in a different electrolyte of Mg[B(hfip)_4_]_2_ in tetraethylene glycol dimethyl ether (G4, tetraglyme) [99]. By applying operando UV/Vis spectroscopy, S_8_, S_6_^2−^, and S_4_^2−^ were identified as the species present in the electrolyte, while S_8_^2−^ and S_3_^•−^ were not detected. A reduction pathway is proposed, with the previously gained insights summarized in Figure 10c. In solvents with a high dielectric permittivity and donor number (e.g., DMSO, DMF, or ACN), the low-charge-density polysulfides S_8_^2−^, S_6_^2−^, and S_3_^•−^ are dominant, while in solvents with a low dielectric permittivity and donor number (G1, G2, G4, THF), the high-charge-density polysulfide S_4_^2−^ is well stabilized.

The intermediate magnesium polysulfides are the main reason for a low Coulombic Efficiency and low cycle performance, which is termed the shuttle effect. The mechanisms by which the shuttle effect diminishes the electrochemical performance of Mg-S batteries can be summarized in three main points:

Reduced Coulombic Efficiency: Coulombic Efficiency refers to the effective utilization of charge during the battery’s charging and discharging processes. Magnesium polysulfides form and dissolve in the electrolyte, with these dissolved polysulfides shuttling between the anode and cathode. This shuttling leads to a portion of the charge in the battery being inefficiently used for energy storage and release, thus reducing the Coulombic Efficiency.

Self-discharge: Self-discharge is a phenomenon where a battery naturally loses its charge when not in use [99]. This process occurs in three stages: (I) the dissolution and reduction into S_6_^2−^ and S_4_^2−^ of S_8_ in the electrolyte, (II) the stabilization of the sulfur concentration and increase in polysulfides (S_6_^2−^/S_4_^2−^), (III) equilibrium of the sulfur and polysulfide (S_8_/S_6_^2−^/S_4_^2−^) concentrations. This self-discharge behavior leads to the loss of active materials in the battery, thereby affecting the battery’s capacity and cycle stability.

Anode Passivation: Due to the low solubility of the sulfides, not all the formed polysulfides can be dissolved, resulting in the formation of insoluble MgS_x_ and MgS at the interface, which poses a significant barrier to further magnetization [68]. This leads to premature termination of discharge, especially at high sulfur/carbon ratios, resulting in lower sulfur utilization.

The extensive research into the sulfur reduction mechanism within Mg-S batteries underscores the pivotal role of the electrolyte composition and solvent dynamics in dictating the battery performance. There is a need for innovative solutions to address challenges such as the shuttle effect and anode passivation, highlighted by the work to enhance Coulombic Efficiency and mitigate self-discharge. This review highlights the key literature in this field, with the aim of advancing the understanding of Mg-S battery chemistry. It has also set a clear direction for the future, with a need to develop electrolyte and battery designs that address these specific challenges, thereby unlocking the potential of Mg-S batteries for high-efficiency, sustainable energy storage.

## 5. Concluding Remarks and Outlook

In this comprehensive review of the development of Mg-S battery electrolytes, we take an in-depth look at the progress, challenges, and future directions in this field. Mg-S batteries have the potential to become a viable alternative to conventional Li-ion systems, offering advantages in terms of energy density, safety, and sustainability. However, their current development is hampered by critical issues such as electrolyte compatibility and anode passivation, as well as being limited by the shuttle effect. Innovations in non-nucleophilic electrolytes, advanced cathode materials, and anode protection strategies are considered key to overcoming these obstacles. This review highlights the need for interdisciplinary research and technological breakthroughs in materials science and electrochemistry to address the complex challenges of Mg-S batteries.

The recent research on Mg-S batteries indicates that, while they have an impressive theoretical energy density of up to 2856 Wh kg^−1^ and rank highly in terms of safety due to the minimal formation of dendrites during charge/discharge, the electrochemical performance of the actual Mg-S batteries is far from ideal. This includes practical metrics such as energy density, cycling stability, and charge/discharge rates, which currently fall significantly short of those in Li-ion batteries. The primary reason for this under-performance, similar to that in Li-S batteries, is the formation and uncontrollability of polysulfides during the charge/discharge cycles. These polysulfides can freely move across the separator between the anode and cathode, a phenomenon known as the “shuttle effect”. This shuttle effect leads to inefficient utilization of some of the battery’s charge in energy storage and release. Moreover, the insoluble polysulfides can form a passivation layer on the electrode surfaces, impeding electrode reactions. These cumulative adverse effects make it challenging for Mg-S batteries to achieve their theoretical electrochemical performance. 

To mitigate the shuttle effect, researchers have employed various strategies, including, but not limited to, 1. Non-Nucleophilic Electrolytes: These do not react with sulfur and thus help reduce the shuttle effect. For instance, non-nucleophilic electrolytes synthesized from hexamethyldisilazane magnesium chloride (HMDSMgCl) and AlCl_3_, as well as organic magnesium borate-based electrolytes containing tetrafluoroborate ammonium ([B(HFP)_4_]^−^), are used. 2. Chloride Additives: Adding an appropriate amount of chloride, such as lithium chloride (LiCl), to non-nucleophilic electrolytes can enhance their stability and electrochemical performance, thereby reducing the shuttle effect. 3. Electrolyte Additives: For example, adding certain ionic liquids, such as PP_14_TFSI, to the electrolyte can improve its conductivity and oxidation stability, which, in turn, helps reduce the shuttle effect. 4. High-Concentration Electrolytes: High-concentration electrolytes can decrease the solubility of the sulfur molecules or polysulfides in the electrolyte, thereby reducing the loss of active material and the shuttle effect. These methods address the shuttle effect according to different mechanisms and have indeed improved the overall performance of Mg-S batteries to a certain extent.

Despite various efforts, breakthrough advancements in the overall performance of Mg-S batteries remain elusive. A significant factor contributing to this stagnation is the lack of a clear understanding of the internal reaction pathways in Mg-S batteries, specifically the sulfur reduction pathways. Although numerous studies have been published investigating the mechanisms of Mg-S batteries, the complexity of the sulfur reduction reactions and limitations in the research methodologies make it challenging for researchers to delineate a complete reaction pathway for each type of electrolyte. There has been some progress, with certain studies proposing relatively comprehensive reaction pathways. However, the authors of these studies also acknowledge discrepancies between their proposed pathways and the characterization results of other research. These inconsistencies might be attributed to the transient nature of some intermediate products or the difficulty of detecting them using traditional methods. This highlights the ongoing challenges in fully understanding and optimizing the electrochemistry of Mg-S batteries.

Non-nucleophilic electrolytes, magnesium borate-based electrolytes (Mg(BPh_4_)_2_, and Mg[B(hfip)_4_]_2_) exhibit a higher capacity, Coulombic Efficiency, and cycle performance compared to the other types of electrolytes, as was shown in Table 1. Benefitting from their high solvation capacity and electrochemical stability, DME and TEG have become the most popular solvents for use with these electrolytes. Looking at the future prospects, there are a number of interesting topics to explore. Additive reduced perylene diimide–ethylenediamine (rPDI) forms a protecting layer on the Mg anode and leads to a high number of stable cycle lives, opening up a promising research prospect. In addition, research into the reaction mechanism, especially in-depth investigations into the mechanisms of the sulfur reduction reaction pathways, is a very important task, and findings in this area will significantly contribute to the mitigation of the shuttle effect, which is key to the future of Mg-S battery use.

In summary, rechargeable Mg-S batteries represent a battery system with immense potential for development, characterized by their high theoretical energy density and proven high safety performance. It is reasonable to anticipate that, in the foreseeable future, a significant breakthrough in the practical electrochemical performance of Mg-S batteries will be achieved.

## Figures and Tables

**Table 1:** Electrolyte systems in Mg-S batteries (according to electrolyte type and in chronological order).

Author/Year	Electrolyte Type	Solute	Solvent	Additives	Coulombic Efficiency [%]	Capacity [mAh g^−1^sulfur]/Current Rate/Cycle Number
H.S. Kim et al., 2011 [52]	Non-nucleophilic Cl-containing	HMDSMgCl	THF	AlCl_3_	95–100	394/no data/2nd
Zhao-Karger, Z et al., 2013 [92]		(HMDS)_2_Mg/ (i-Pr_2_N)_2_Mg	THF/Diglyme/Tetraglyme	AlCl_3_	97–98	90/10 mA g^−1^/30th
Zhao-Karger et al., 2014 [55]		(HMDS)_2_Mg	Diglyme/Tetraglyme	AlCl_3_+ PP_14_TFSI	100	150/0.01 C/20th (PVDF, diglyme) 200/0.01 C/20th (CMC, diglyme) 250/0.01 C/20th (PVDF, tetraglyme) 260/0.01 C/20th (CMC, tetraglyme)
Gao et al., 2015 [17]		(HMDS)_2_Mg	No Data	AlCl_3_ + LiTFSI	92	1000/0.03 C/30th
Du et al., 2017 [71]		B(HFP)_3_/OMBB	DME	MgCl_2_	80.4% (100th)	1000/0.1 C/100th
Zhao et al., 2019 [93]		Magnesium bis(diisopropyl)amide	THF	AlCl_3_ + LiCl	94	400/0.04 C/100th
Yang et al., 2018 [53]		Mg(CF_3_SO_3_)_2_ + anthracene	THF + Tetraglyme	AlCl_3_ + LiCl/ LiCF_3_SO_3_	100	300/0.05 C/55th 400/0.05 C/55th
Sun et al., 2021 [94]		Mg(TFSI)_2_	DME	MgCl_2_ + rPDI	99.4 98	110 (1 mg cm^−2^ loading)/15 C/1000th 100 (10 mg cm^−2^ loading)/1 C/200th
Xu et al., 2019 [95]		Mg(BPh_4_)_2_	PYR14TFSI	YCl_3_	98.7	1000/0.04 C/50th
Li et al., 2016 [77]	Non-nucleophilic Cl-free	[Mg(THF)_6_]^2+^	PYR14TFSI + THF	No data	100	63/0.02 C/20th
Zhao-Karger, Z et al., 2017 [51]		Mg[B(hfip)_4_]_2_	DME + TEG	No data	100	200/0.1 C/100th
Zhao-Karger, Z et al., 2018 [84]		Mg[B(hfip)_4_]_2_	DME	No data	100	200/0.1 C/100th
Zhang et al., 2017 [50]		THFPB	DME	MgF_2_	100	900/0.03 C/30th
Ren et al., 2021 [60]		MBA + AlF_3_	THF	LiTFSI + PP_14_TFSI	100	260/0.2 C/70th
Zeng et al., 2017 [96]	Nucleophilic	(PhMgCl)_2_	THF	AlCl_3_	100	300/0.005 C/40th
Wang et al., 2018 [54]		(PhMgCl)_2_	THF	AlCl_3_	100	368/0.1 C/200th

## Data Availability

All data generated or analyzed during this study are included in the article.
